# Laparoscopic fundoplication and new aspects of neural anatomy at the oesophagogastric junction

**DOI:** 10.1002/bjs5.50271

**Published:** 2020-03-05

**Authors:** P. Gehwolf, O. Renz, E. Brenner, B. Cardini, A. Lorenz, H. Wykypiel

**Affiliations:** ^1^ Department of Visceral Transplant and Thoracic Surgery, Centre for Operative Medicine Innsbruck Austria; ^2^ Department of Anatomy, Histology and Embryology, Division of Clinical and Functional Anatomy Medical University of Innsbruck Innsbruck Austria

## Abstract

**Background:**

In fundoplication, mobilization of the distal oesophagus and proximal stomach is essential to obtain a sufficient tension‐free intra‐abdominal oesophageal length for creation of an efficient antireflux barrier. Most surgical literature and anatomical illustrations do not describe nerve branches running from the diaphragm to the stomach. After observing small nerve branches at laparoscopic fundoplication, penetrating the left crus of the diaphragm lateral to the hiatus and apparently running into the stomach, an anatomical cadaver study was undertaken to identify the origin and target organ of these nerves.

**Methods:**

Fifty‐three human cadavers (23 men, 30 women; age range 35–103 years) were dissected with special attention to the nerves that penetrate the left crus of the diaphragm. The entire course of these nerves was documented with standardized drawings and photos.

**Results:**

Small nerve branches penetrating the diaphragm lateral to the left crus of the hiatus were found in 17 (32 per cent) of the 53 cadavers. In 14 of these 17 cadavers, one or two splanchnic nerve branches were identified, and in ten of the 17 the nerve branches were found to be phrenic nerves. In seven of these 17 cadavers, two different nerve branches were found and assigned to both splanchnic and phrenic nerves.

**Conclusion:**

Nerves penetrating the left crus with splanchnic origin or phrenic origin have been identified. Their function remains unclear and their relationship to postfundoplication symptoms remains to be determined.

## Graphical abstract

Small nerve branches penetrating the left crus of the diaphragm lateral to the hiatus, presumably running into the stomach, were observed during surgery. These nerve branches were allocated to the splanchnic and phrenic nerves. The origins and target organs of these nerves were identified. Their role in postfundoplication complaints is discussed.

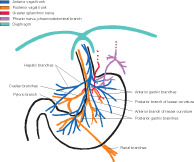

Nerves that might influence outcome.

## Abstract (es)

**Antecedentes:**

A la hora de realizar una fundoplicatura, la movilización del esófago distal y del estómago proximal es esencial para obtener una longitud de esófago intraabdominal suficiente y sin tensión para crear una barrera antirreflujo eficiente. La mayoría de la literatura quirúrgica y de las ilustraciones anatómicas no describen unas ramas nerviosas que discurren desde el diafragma al estómago. Tras observar pequeñas ramas nerviosas durante la realización de una fundoplicatura laparoscópica que penetran la crura izquierda del diafragma lateral al hiato y que aparentemente discurren hacia el estómago, se llevó a cabo un estudio anatómico en cadáver para identificar el origen y el órgano diana de estos nervios.

**Métodos:**

Se diseccionaron 53 cadáveres humanos (23 varones, 30 mujeres, rango de edad: 35‐103 años) con especial atención hacia los nervios que penetran la crura izquierda del diafragma. Se documentó el recorrido completo de estos nervios con fotos y dibujos de una forma estandarizada.

**Resultados:**

En 17 (32%) de 53 cadáveres se hallaron pequeñas ramas nerviosas que penetraban el diafragma lateral a la crura izquierda del hiato. En 14 cadáveres (de los 17) se identificaron una o dos ramas nerviosas esplácnicas, y en 10 de los 17 cadáveres las ramas nerviosas que se hallaron resultaron ser nervios frénicos. En 7 de estos 17 cadáveres, se hallaron dos ramas nerviosas diferentes y se asignaron a ambos nervios, esplácnicos y frénicos.

**Conclusión:**

Se han identificado los nervios que penetran la crura izquierda con un origen esplácnico y frénico. Sus funciones están por aclarar, así como su relación con los síntomas que aparecen tras la fundoplicatura.

## Introduction

All forms of fundoplication rely on the creation of a sufficient intra‐abdominal oesophageal length to ensure a gastro‐oesophageal wrap with restoration of a high‐pressure zone within the abdomen. Separation of the abdominal compartment from the thoracic compartment by a hiatoplasty helps to retain the fundoplication in the abdomen for a competent antireflux barrier^1^, ^2^, ^3^, ^4^, ^5^.

During posterior laparoscopic fundoplication, mobilization of the distal oesophagus and proximal stomach is necessary to obtain sufficient intra‐abdominal oesophageal length. To achieve this, the gastrophrenic ligament must be dissected and the oesophagus cleared from the diaphragmatic crura over a sufficient length to facilitate crural closure and allow the mobilized fundus to be passed between the repaired hiatus and the posterior oesophagus.

A variety of side‐effects following fundoplication have been described, such as dysphagia, postprandial bloating, inability to belch and inability to vomit, that are generally attributed to hiatal narrowing or ‘supercompetence’ of the high‐pressure zone^3^, ^6^. Their pathophysiology is, however, not fully understood, but denervation of the stomach by mobilizing the fundus has been shown to enhance ‘wind‐related’ problems^6^.

During fundoplication, small nerve branches penetrating the left crus of the hiatus and thought to be entering the stomach have been recognized regularly. Their course (from lateral to medial) suggested that they could be branches of the splanchnic or phrenic nerves rather than vagal branches. Based on these observations, the frequency and distribution of these nerves was investigated.

After conducting a MEDLINE search using five different key phrases (splanchnic nerve, fundoplication, postfundoplication symptoms, bloating, GERD), personal contact with experts (surgeons, gastroenterologists and anatomists), and discussion at national and international conferences, it became clear that there was little awareness of the existence and nature of these nerve branches. MEDLINE and Google Scholar searches revealed a single report by a surgeon^7^, four human anatomical studies^8^, ^9^, ^10^, ^11^ and five animal studies^12^, ^13^, ^14^, ^15^, ^16^.

The aim of this cadaver study was to confirm the existence of these nerves and to describe their anatomical background (origin, course and target site).

## Methods

Anatomical dissections were performed on 53 Caucasian cadavers (30 women and 23 men; age range 35–103 years) to identify nerves passing between the left crus and the cardia, and to clarify their origin and target structure. Bodies were donated to the Division of Clinical and Functional Anatomy of the Medical University of Innsbruck, where donors had provided informed consent for their later use for scientific and educational purposes^17^. All cadavers were preserved by administration of an arterial injection of a formaldehyde–phenol solution and immersion in phenolic acid and water for 1–3 months^18^.

**Figure 1:**
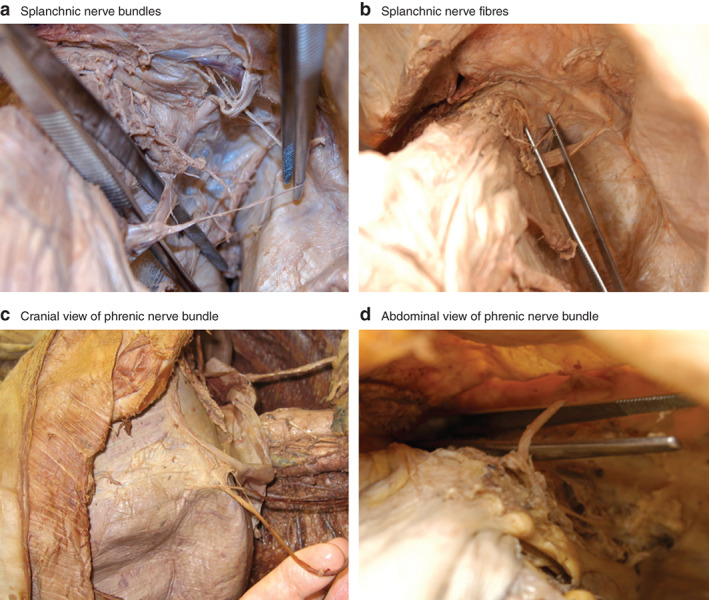
Penetration site and course of splanchnic and phrenic nerve bundles **a** Bundles and **b** fibres of the splanchnic nerve; **c** cranial and **d** abdominal view of phrenic nerve bundle.

To achieve sufficient exposure, a transection was performed in the anterior axillary line through the clavicles, thorax and abdominal wall, after which parts of the thoracic and abdominal viscera were removed. The right and left hemidiaphragms were trimmed to a level superior to the point of passage of the thoracic splanchnic nerves through the crura. After removal of the parietal pleura and dissection of the thoracic and abdominal sympathetic chain, as well as the preaortic ganglia, the greater, lesser and least thoracic splanchnic nerves were dissected free in their full length from their ganglionic origins to their merging with the target structures. The course of the nerves was plotted and documented in photos (*Fig*. 1). The point of penetration through the diaphragm was displayed as a clockface view, looking cephalad. The distance between the penetration site and the edge of the hiatus was measured.

## Results

Small nerve branches penetrating the diaphragm lateral to the left crus of the hiatus were found in 17 (32 per cent) of the 53 cadavers (*Table* 1). In 14 of the 17 cadavers, one or two nerve branches could be allocated to the splanchnic nerves. In ten cadavers, the nerve branches were allocated to the phrenic nerve. In seven of the 17 cadavers, two different nerve branches were found, and assigned to both splanchnic and phrenic nerves.

**Table 1:** Penetration site (in clockface position) of splanchnic and phrenic nerve fibres, distance from penetration site to the left crus, and target organ in 17 cadavers

Cadaver	Sex	Age (years)	Splanchnic nerve	Penetration distance (cm)	Position (o'clock)	Target	Phrenic nerve	Position (o'clock)	Penetration distance (cm)	Target
1	F	101	1	2	4	Fundus	No			
2	F	62	1	5	4	Fundus, coeliac ganglion	Yes	3	9	Fundus
3	F	89	1	1·5	4	Fundus, coeliac ganglion	Yes	4	7·5	Fundus
4	F	92	1	3	3	Fundus, coeliac ganglion	Yes	3	5	Fundus
5	M	62	1	1	4	Coeliac ganglion	Yes	4	10	Fundus
6	F	82	1	3	4	Fundus, corpus	Yes	4	5	Fundus
7	F	89	1	7·5	4	Fundus, corpus	Yes	?	?	?
8	M	57	No				Yes	4	12	Fundus
9	M	58	1	1	3	Corpus, coeliac ganglion	No			
10	F	103	1	2	2	Corpus, coeliac ganglion	No			
11	F	92	No				Yes	4	9	Fundus
12	F	54	1	3·5	4	Fundus, coeliac ganglion	Yes	4	5	Fundus, corpus
13	M	58	1	1·5	4	Fundus, coeliac ganglion, corpus	No			
14	F	92	2	3; 1·5	4; 4	Fundus; fundus	No (no vagus)			
15	F	93	2	3; 4	4; 5	Fundus, coeliac ganglion, corpus	No			
16	F	93	1	1	5	Fundus	No			
17	F	85	No				Yes	4	10	Fundus

In 14 of the 17 cadavers with either phrenic and splanchnic nerves or with single splanchnic nerves, the splanchnic nerves penetrated the left crus at 4 (range 2–5) o'clock, as seen from the abdomen, at a median distance of 2·5 (1–7·5) cm from the edge of the hiatus to the penetration site. In two of these 14 cadavers with splanchnic nerves, two splanchnic nerve branches were observed.

The target site of the splanchnic nerves in these 14 cadavers was the fundus in 11 and the corpus of the stomach in six. In nine of the 14 cadavers, additional splanchnic branches ran into the coeliac ganglion.

In ten cadavers in which a branch of the phrenic nerve was identified, these nerves penetrated the left crus at 4 (range 3–4) o'clock, as seen from the abdomen. The distance from the edge of the hiatus to the penetration site was a median of 9 (range 5–12) cm. In nine of these ten cadavers, the target site was the fundus. In one cadaver, the course of the nerve could not be mapped accurately.

**Figure 2:**
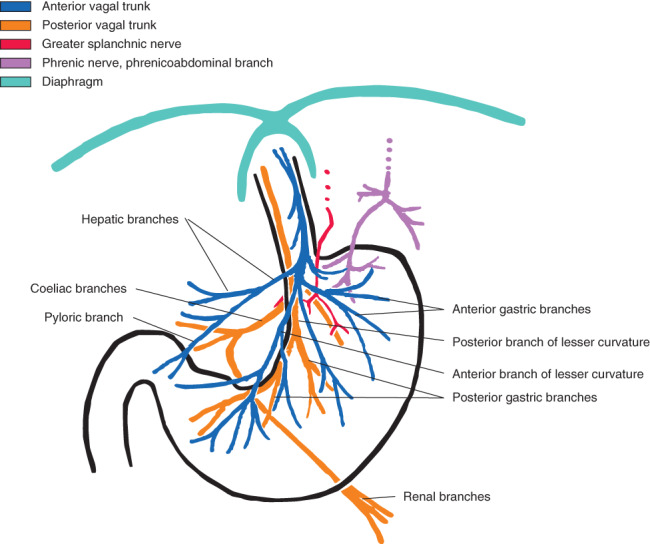
Graphic depiction of the branches of the vagus nerve along the stomach The described courses of the observed splanchnic and phrenic nerves are set in relation to the vagus nerves.

*Fig*. 2 shows the course and origin of the documented nerve bundles in relation to the vagus nerves.

## Discussion

This study has verified the existence of nerves seen at laparoscopic surgery lateral to the hiatus in about one in three cadaveric dissections; these nerves appear to be branches of either splanchnic or phrenic nerves. These nerve bundles have been described only occasionally^7^, ^9^, ^10^, ^11^ and without evidence for their function in humans.

The splanchnic nerves contain both visceral afferent and efferent fibres^19^, ^20^. In animal studies, transection of the splanchnic nerves leads to increased intragastric pressure, whereas splanchnic nerve stimulation results in short‐term relaxation of the stomach with rapid return to resting pressure^15^. Splanchnic nerves are considered to play an important role in gastric contraction and determination of gastric pressure^14^, ^15^, ^21^. Mechanoreceptors via afferent splanchnic fibres or efferent stimulation are thought to influence satiety, gastric emptying, nausea and bloating^12^, ^22^, ^23^.

There is little evidence to support a specific function for phrenic nerve fibres, although a single Japanese study^24^ found that stimulation of the phrenic nerve in dogs enhanced gastric contractions.

Avoidance of excessive division of the gastrophrenic ligament (including short gastric vessels), which inevitably divides splanchnic fibres, has been shown to be associated with fewer ‘wind‐related’ problems^6^, ^25^. Laparoscopic anterior fundoplication, with less need for mobilization of the fundus, seems to be associated with less dysphagia than posterior fundoplications, but with no difference in inability to belch, gas bloating or patient satisfaction^25^, ^26^, ^27^. To what extent sparing of splanchnic nerve fibres influences these symptoms remains unknown.

This study has limitations. During operation at the hiatus, the frequency of the above‐described nerves was not sought systematically. Their frequency in cadaveric dissections may be different from recognition at laparoscopy. There were insufficient cadavers to ascertain changes in the ability to identify these nerves based on age, sex or body habitus. Although anatomy demonstrators were regularly present, dissections performed by medical students during their anatomy course may well have caused destruction of some structures, so that the numbers of nerves found may have been underestimated. Histological assessment of these nerves or complete neuroanatomical processing was not performed, as the primary aim was to confirm the existence of these nerves.

Further studies are needed for accurate enumeration and full characterization of subdiaphragmatic splanchnic or phrenic nerve bundles. It might then be appropriate to investigate any relationship with postfundoplication symptoms.
